# Chronotolerance study of the antiepileptic drug valproic acid in mice

**DOI:** 10.1186/1740-3391-10-3

**Published:** 2012-05-10

**Authors:** Wafa Ben-Cherif, Ichrak Dridi, Karim Aouam, Mossadok Ben-Attia, Alain Reinberg, Naceur A Boughattas

**Affiliations:** 1 Laboratoire de Pharmacologie, Faculté de Médecine, Université de Monastir, Monastir, Tunisia; 2 Laboratoire de Biosurveillance de l’Environnement, Faculté des Sciences de Bizerte, Université de Carthage, Carthage, 7021, Zarzouna, Tunisia; 3 Unité de Chronobiologie, Fondation A.de Rothschild, 75940, Paris, cedex 19, France

**Keywords:** Valproic acid, Chronotolerance, Circadian rhythm, Mice

## Abstract

**Background:**

Valproic acid (VPA) is an antiepileptic drug widely used for the treatment of absence seizures and generalized tonic-clonic seizures. The present work aims to study whether VPA-induced toxicity varies according to the dosing-time in the 24 hour-scale.

**Methods:**

The influence of dosing-time on tolerance to VPA was investigated in 120 male Swiss mice synchronized under a light-dark cycle (12:12). The mean VPA lethal dose was first determined to be 850 ± 0.2 mg/kg, *i.p.*. Such a dose was administered by *i.p.* route to a total of 90 mice divided in six circadian stages [1, 5, 9, 13, 17 and 21 Hours After Light Onset (HALO)] (15 mice/circadian time); 30 mice were used as control (5 mice / circadian time).

**Results:**

The surviving treated mice exhibited a significant circadian variation in rectal temperature and body weight loss (p < 0.001). The least rectal temperature change and body weight loss occurred when VPA was injected at 9 HALO. Drug dosing at 9 HALO resulted in -9 % weight loss whereas drug dosing at 17 HALO was -15 % (Ø = 20.3 HALO ± 1.1 h, p ≤ 0.0001). Lethal toxicity also varied according to circadian dosing-time (χ^2^ = 42.1, p < 0.0001). The highest (60 %) and the lowest (6.67 %) survival rates were observed at 9 HALO and 17 HALO respectively. Cosinor analyses validated a significant circadian rhythm in survival duration with an acrophase at 8.4 HALO ± 0.75 h (p < 0.001).

**Conclusions:**

With regards to these data the optimal tolerance to VPA occurred when the drug was administered in the second half of the light-rest span of mice which is physiologically analogous to the second half of the night for human patients.

## Background

Biological organisms are highly organized according to one or more internal biological clocks. The master mammalian biological clock is located in the hypothalamic suprachiasmatic nuclei (SCN) which imposes its temporal structure on the organism via neural and endocrine outputs [1,2]. The SCN harbors the master circadian clock that coordinates most aspects of behavior and physiology [3,4]. This endogenous clock is responsible for biological rhythms which are influenced by synchronizing factors such as light, temperature and food intake [5].

Biological responses to various drugs follow circadian rhythms in experimental animals as well as human beings. Many drugs vary in potency and / or toxicity associated with the rhythmicity of biochemical, physiological, and behavioral processes [6,7]. Theoretically, it has been argued that drug administration at certain times of the day should improve the outcome of pharmacotherapy. Identification of rhythms in animal models helps provide an optimal dosing-time and suggests guidelines to a potential chronotherapy. The dosing of a medication at the proper biological time with reference to circadian rhythms can result in modulation of its efficiency or its toxicity as demonstrated, in particular, for the anticancer agents used in human chemotherapy [8].

Epilepsy is one of the most common diseases of the brain and the chronopharmacological studies of antiepileptic drugs are of considerable importance for optimizing therapeutic tolerance in patients and reduce important adverse effects of these drugs [9,10]. Valproic acid (VPA) is a short branched-chain fatty acid that was serendipitously discovered in 1963 to prevent pentylenetetrazole (PTZ)-induced seizures [11,12]. Since then, its therapeutic role has expanded to include migraine and bipolar disorder prophylaxis, as well as a large list of potential new roles, including Alzheimer’s disease, cancer and HIV treatment [13]. This drug is today one of the major antiepileptic drugs having a broad-spectrum activity against different kinds of epilepsy and is generally regarded as a first line drug for the treatment of primary generalized epilepsy [14]. A better understanding of the mechanisms involved in VPA toxicity should help to further optimize the chronotherapy of epilepsy.

In this context, the aim of the present study was to document an effect of dosing-time upon murine tolerance to VPA. The circadian variation in rectal temperature, survival and body weight change, were used as toxicity endpoints.

## Methods

### Animals and entrainment

A total of 120 ten-week-old Swiss albino mice (SIPHAT, Foundouk Choucha Street, Ben Arous 2013, Tunisia) were used in this study. The animals were housed five per cage and acclimated to conditions of our animal research facility for at least 3 weeks prior to the beginning of experiments. During this period, mice were entrained in two air-conditioned rooms specially designed for chronobiological investigations under a lighting regimen consisting of an alternation of 12 hr of light (L) and 12 hr of darkness (D) (LD 12:12). Light-dark cycle regime was inversed between the 2 rooms (Room 1: L from 7 to 19; Room 2: L from 19 to 7) in order to allow the exploration of circadian times during the day [15]. The room temperature was maintained at 22 ± 2°C and the relative humidity was about 50 - 60%. During all experiments, a standard diet and water were provided ad libitum. The animals were randomly divided into various groups of 20 mice each at 6 circadian stages denoted as 1, 5, 9, 13, 17, and 21 Hours After Light Onset (HALO).

All experiments were performed according to the guidelines of care and use of laboratory animals [16]. In the present study, the entrainment is assessed by the circadian rhythmicity in rectal temperature, the acrophase (peak time) used as a marker rhythm index [17].

### Drug

The VPA solution was freshly prepared prior to each study by adding an adequate volume of sterilized physiological saline with few drops of Tween 80. The dose used for toxicity study was 850 mg/ kg, which corresponds to a 85 mg / ml concentration. Each dose was administered intraperitonally *(i.p.)* to mice in a fixed fluid volume (10 mL / kg, body weight).

### Study design

A preliminary study was conducted to determine the lethal dose 50 (the dose inducing 50% of lethality in mice). A total of 90 mice were used for this preliminary study (15 mice for each circadian stage). Different doses of VPA were prepared following a geometric progression with a ratio of 1.2 between each two successive doses (550, 660, 792, 950 and 1140 mg / kg). Ninety mice were used in this study and the drug was *i.p.* administrated at a single fixed time (11:00 h local time, i.e., 4 HALO) during the diurnal (rest) span to minimize the influence of circadian changes. The Miller and Tainter method was used to estimate the lethal toxicity and the LD_50_ was estimated from curves expressed as percentage (%) of mortality (converted to a probit scale) against the log-dosage of VPA [18].

To explore the three end-points of toxicity investigated: rectal temperature, survival rate and body weight loss, a total of 120 mice were used. The mice were treated at each of the six selected circadian stages. Thirty control mice (5 mice / time point) received sterile distilled water mixed with Tween 80. Ninety treated mice (15 mice / time point) received by *i.p.* route a potentially lethal dose (850 mg / kg, *b.w.,* which corresponds to the LD_50_).

After treatment, mortality was recorded thereafter daily throughout a 30-day span. Temperature and body weight were assessed every 2 days, the registration of the temperature and weight was done at the circadian time which corresponds to the drug injection time. Body weight was measured by a high-precision balance (RADWAG, WPS 360/C/2) and rectal temperature was assessed with a digital thermometer (OMRON Ecosmart, Holt 55005). Survival rate was determined for each group at the end of the study. Body weight loss was computed as the percentage relative to the initial body weight prior to treatment [19].

### Statistical analysis

Means and one standard error of the mean (S.E.M) were computed for each circadian dosage-time. For quantitative data, one-way analysis of variance (ANOVA) was used to test the significance of differences over time. The differences in survival rate were analyzed with the χ^2^ test. Both conventional and chronobiological statistical methods were used to validate significant temporal changes and rhythms [20].

The cosinor was used to evaluate the presence of 24-hour rhythmicity in the effects of injection time on the recorded variables (body temperature, body weight and mortality). For a given period, a rhythm was characterized by three parameters: the mesor (M) is the 24 h rhythm adjusted mean, the amplitude (A) is the difference between peak and trough of the best-fitting cosine function and the acrophase (Ø) is the peak time (with light onset 0 HALO used as phase reference) [21].

All rhythm characteristics were obtained with their 95 % confidence limits. In principle, a rhythm is detected when A is different from zero (non-null amplitude as verified by F test of the variance accounted by the fit of the time series data to cosine curve of a given period versus that accounted for by a straight line fit to the time series data at p < 0.05) [22].

## Results

### Entrainment of mice

In this study, a circadian rhythm in rectal temperature (computed for the combined data of the different HALO groups) was validated by the cosinor analysis on day 1 (p < 0.0001). The acrophase of this 24 h rhythm occurred near the mid-half of the dark-activity span (Ø = 16.2 HALO ± 0.6 h). The characteristics of the 24 h pattern in rectal temperature confirmed the physiological entrainment of mice to the environmental LD schedule. Such entrainment allows the use of the circadian acrophase of rectal temperature as a marker for the dosing time-dependent differences in VPA associated toxicity.

### Rectal temperature

**Figure 1:**
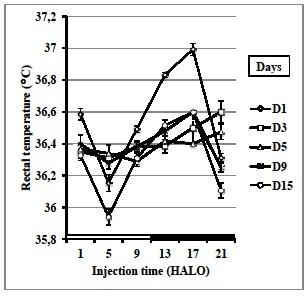
**Rectal temperature (°C) variation according to the circadian time (1, 5, 9, 13, 17 and 21 HALO) of VPA administration (850 mg / kg,*i.p.*) in a total of 90 mice on day 1 before treatment, and on days 3, 5, 9 and 15 following treatment in surviving mice.** Differences related to both dosing-time and sampling-day were statistically validated by one-way ANOVA (p ≤ 0.01).

Rectal temperature changes were only dosing-time dependent (ANOVA with p < 0.01), but not sampling-day dependent. The mean rectal temperature decreased from the pretreatment baseline in a non-statistically significant manner after VPA injection. Rectal temperature varied significantly according to the administration time of VPA during all days of the study (Figure 1). A physiological circadian rhythm in rectal temperature of treated mice was detected by the cosinor analysis (p < 0.0001) and its acrophase was located in the first half of the dark span without a shift in timing compared to pretreatment control day 1. The acrophase on days 3, 5, 9 and 15 were respectively 14.4 HALO ± 0.8 h, 20.1 HALO ± 0.6, 15.6 HALO ± 0.9 and 16.2 HALO ± 0.4 h. Furthermore, there was no significant variation in mesor throughout the study. Regarding the amplitude, this parameter varies significantly depending on the day following treatment (ANOVA p < 0.01). The amplitude on day 1 was 0.37 ± 0.07, it decreases significantly at days 3 and 5 (respectively 0.1 ± 0.02 and 0.07 ± 0.01), from the day 9, the amplitude increased to 0.12 ± 0.02. On day 15, the amplitude reached 0.35 ± 0.03 which was not statistically different from day 1.

### Body weight changes

Body weight varied across the time of the day. On Day 1, before treatment, body weight varied between 29.61 g and 25.14 g at respectively 1 and 13 HALO. A circadian rhythm was detected by cosinor with an acrophase located at 1.53 HALO ± 0.68 h. In Mice surviving for 15 days, the circadian rhythm in body weight remained present (p < 0.001) with a shift of the circadian acrophase location from day 5 ( Ø = 22.87 HALO ± 0.886 h).

**Figure 2:**
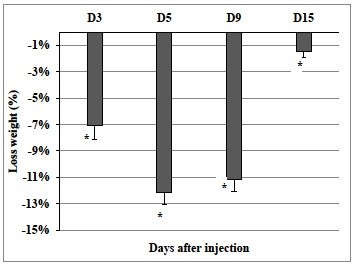
**Body weight loss variation (%) according to the days after VPA-administration in a total of 90 mice.** Results are expressed as mean percent change ± S.D and were statistically validated by one-way ANOVA (p ≤0.01).

A statistically significant loss of body weight, as function of sampling day, was also observed (ANOVA; p < 0.01). From day 7, surviving mice began to recover weight. With reference to the pretreatment baseline on day 1, the mean losses (denoted by a negative sign) were -7.1% on day 3, -12.2% on day 5, -11.2% on day 9, and -1.4% on day 15 (Figure 2).

**Figure 3:**
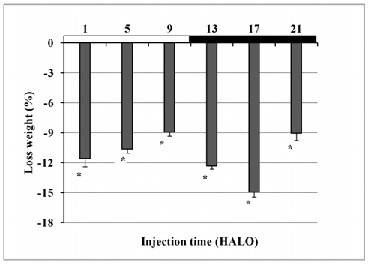
**Body weight loss variation (%) according to the circadian dosing-time of VPA in a total of 90 mice. Results are expressed as mean percent change ± S.D.** Statistically significant differences between mean values at least and highest body weight losses were validated by one-way ANOVA (p ≤ 0.01).

Body weight loss varied according to dosing-time of VPA (ANOVA p < 0.01) (Figure 3). It was minimal (-8.9%) in the group injected at 9 HALO [light (rest) span] and maximal (-14.9%) in the group treated at 17 HALO, and peak-trough differences were statistically significant. The cosinor analysis also documented a circadian rhythm in body weight loss (p < 0.001). The estimated treatment time corresponding to maximal weight loss (acrophase) was located at 20.3 HALO ± 1.2 h.

### Survival rate

**Figure 4:**
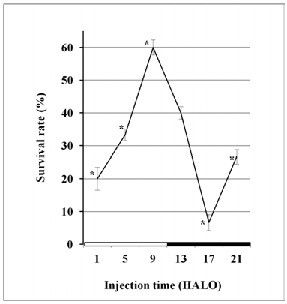
**Circadian variation in tolerance to VPA (850 mg/kg,*i.p.*) as assessed by the survival rate at each of six circadian dosing-times (1, 5, 9, 13, 17, and 21 HALO) in a total of 90 mice.** Results are expressed as mean percent change ± S.D and were statistically validated by χ 2 test (p ≤ 0.001).

A general CNS depressant response with decreased spontaneous activity and alertness was observed in all animals. In addition to the behavioral toxicity described above, we observed tremor, subdued behavior, prostration, respiratory distress, and finally death that was preceded by convulsions. Deaths occurred between days 1 and 4 following VPA injection. Subsequently, no death occurred until day 30 when the study was completed. Over all dosing times, the mean survival rate was 31%. However, lethal toxicity varied largely according to the circadian dosing-time (p ≤ 0.0001). Survival rate differed as a function of VPA dosing-time, the difference being validated by χ^2^ test (χ^2^ = 42.1, p < 0.0001) and by the Cosinor analysis. The highest survival rate (60%) occurred when VPA was injected at 9 HALO, while the lowest survival rate (7%) occurred when VPA was given at 17 HALO (middle of the dark-activity span of mice) (Figure 4). A circadian rhythm in survival was detected by cosinor (p < 0.0001) with an acrophase Ø localized at 9 HALO ± 0.6 h

## Discussion

The field of chronobiology has advanced greatly in recent decades, achieving important breakthroughs in research in nearly all areas especially in chronopharmacology. The temporal changes in drug effects include variations both in the desired (chronoeffectiveness) and undesired (chronotoxicity) effects. Although the dosage of drug is the same, the effects vary depending on the time of administration. This suggests the presence of a rhythm in drug susceptibility in tissues and / or in pharmacokinetics [5]. Numerous studies in animals as well as clinical studies have provided convincing evidence that the drugs’ side effects can be modified by the circadian time and/or the timing of drug administration within 24 h of a day [23]. The choice of the most appropriate time of the day for drug administration (i.e., the circadian phase at which the drug is administered) may help to achieve rational chronotherapeutics of epilepsy [24], as it was the case of other diseases such as asthma, cancer and hypertension in both experimental and clinical studies [7].

The obtained results in the present study suggest the importance of the circadian time at which VPA is administered in the experimental studies in mice. In the current study, animal entrainment was verified using rectal temperature as a marker rhythm. The temperature circadian rhythm constitutes one of the most important toxicity end-points in animal experiments with reference to its reliability [25]. Like the findings of earlier reports, the peak in rectal temperature occurred almost in the middle of the dark (activity) span of mice. This peak location coincides with the occurrence of the highest physical activity in mice, i.e. during the dark span [8,17]. Rectal temperature varied as function of VPA dosing-time. A slight but not significant hypothermia was observed in VPA-treated mice. This data confirms the clinical results obtained in a study which demonstrated that a VPA treatment in children induces hypothermia and thermoregulatory dysfunctions [26]. Despite this slight temperature decrease, the circadian rhythm in rectal temperature was not altered. The acrophase was maintained at the same circadian time as that of control mice with no significant decrease in mesor.

A significant circadian rhythm was demonstrated for acute toxicity measured by VPA induced-mortality. Optimal tolerance for valproic acid was achieved when this drug was given in the second half of rest span of mice (9HALO), while the maximum toxicity was found when VPA was administered in mice at 17 HALO; time which corresponds to the peak of murine rectal temperature. Whatever the index chosen to assess the murine tolerance, the circadian variation in survival rate and body weight loss were similar. The highest survival rate (60%) as well as the least body weight loss (-8.94%) occurred when VPA was administered at 9 HALO.

It is well documented that seizures in experimental and clinical epilepsy demonstrate circadian or sleep-wake state regulation in a syndrome-dependent form [27]. Day-night variation in seizure susceptibility has been attributed to the alterations in tissue responsiveness secondary to biochemical changes that occur throughout the photoperiod [9]. These results suggest that these rhythms must be regarded as one of the most important factors for investigation of various brain functions. In addition, studies of the mechanisms of the rhythm in drug efficacy can be used to investigate the causal mechanisms of drug action and brain function. Seizure thresholds in response to convulsive treatments also vary with the time of day. Based on the available information, circadian patterns of seizures may be used for diagnostic and therapeutic purposes; in addition to the variation in neuronal excitability, chronological changes in pharmacodynamics and pharmacokinetics may play a role in therapeutic efficacy of pharmacotherapy [28]. In this context, VPA embryotoxicity in rats has been shown to follow a circadian pattern [29]; otherwise a constant rate administration of subcutaneous VPA in mice through implanted osmotic minipumps demonstrated a significant circadian rhythm of VPA levels [11]. In the human brain, VPA interacts with the activity of the neurotransmitter Gamma Amino Butyrate (GABA) by potentiating the GABA inhibitory activity through several mechanisms including inhibition of GABA degradation, inhibition of GABA Transaminobutyratre (GABA-T), increased GABA synthesis and decreased turnover [30]. Moreover, VPA attenuates N-Methyl-D-Aspartate-mediated excitation and blocks Na^+^ and Ca^2+^ channels [31,32]. The site of VPA action is closely associated with the inhibitory central nervous system including the GABA system [33]. The presence of a circadian rhythm has been reported for neurotransmitters and their related substances such as GABA; indeed a 24-hour period rhythm is observed for GABA [34]. The enzyme activities related to neurotransmitters also have a circadian rhythm.A rhythm has also been reported for the enzyme activity glutamic acid decarboxylase involved in GABA synthesis.

The most typically undesired effects of VPA administration in mice are subdued behavior and respiratory distress which were observed 3 to 5 minutes after drug administration [35]. Similar experimental signs were described in a study about the toxicity induced by VPA overdose. Naloxone, a morphine antagonist, has been demonstrated to antagonize also the central depressant effect induced by VPA and that by GABA in the brain [36]. The rhythm for several biogenic amines in the brain also plays a role in the rhythm of toxicity of drugs acting on the autonomic and central nervous system. In addition to producing sedation and hypnosis, the central inhibitory drugs such as barbiturates are generally known to affect respiration and body temperature. Respiratory and thermoregulatory function show a circadian rhythm associated with the autonomic nervous system [37]. The mechanisms underlying the action of VPA may differ between the effectiveness and the toxicity. Although VPA is thought to act on the inhibitory central nervous system including GABA, the effective VPA dose does not cause respiratory failure. However, the toxic VPA dose acts on the system controlling respiration and produces respiratory failure. Respiratory failure induced by the toxic VPA dose may be attributed to the central inhibitory effect. Therefore, the factors described above may be related to the VPA-induced chronotoxicity [38].

The choice of the most appropriate time of the day for drug administration may help to achieve rational chronotherapeutics of some drugs including VPA in clinical use [39,40]. The most important finding of our study is that the VPA chronotoxicity is maximized when it was administered during nearly the dead center of the animals’activity and minimized when it was administered during the latter half of the animals’s sleep phase. This result is very important since VPA is a drug which is always administered during the diurnal phase in humans (activity span), it seems necessary here to remember that Swiss albino mice are nocturnal rodent, being more active and awake during the dark phase when maintained on a light/dark cycle, which is the case in our study.

A better understanding of periodicity of epileptic phenomena is very important for epilepsy chronotherapy. So far, only limited information on chronotherapy in patients with epilepsy is available. The use of epilepsy chronotherapy will be contingent upon the amount of knowledge generated from future epilepsy chronobiology research.

## Competing interests

The authors declare that they have no competing interests.

## Authors’ contributions

WBC conceived of the study, conducted data analysis, drafted the manuscript and with ID carried out the experiments. AR, MBA and NB contributed to reviewing the manuscript and interpretation of the study. NB and KA provided samples and clinical information. All authors read and approved the final version of the manuscript.
